# Human Infection with Influenza A(H7N9) Virus during 3 Major Epidemic Waves, China, 2013–2015

**DOI:** 10.3201/eid2206.151752

**Published:** 2016-06

**Authors:** Peng Wu, Zhibin Peng, Vicky J. Fang, Luzhao Feng, Tim K. Tsang, Hui Jiang, Eric H.Y. Lau, Juan Yang, Jiandong Zheng, Ying Qin, Zhongjie Li, Gabriel M. Leung, Hongjie Yu, Benjamin J. Cowling

**Affiliations:** World Health Organization Collaborating Centre for Infectious Disease Epidemiology and Control, School of Public Health, Li Ka Shing Faculty of Medicine, University of Hong Kong, Hong Kong, China (P. Wu, V.J. Fang, T.K. Tsang, E.H.Y. Lau, G.M. Leung, B.J. Cowling);; Division of Infectious Disease, Key Laboratory of Surveillance and Early-warning on Infectious Disease, Chinese Center for Disease Control and Prevention, Beijing, China (Z. Peng, L. Feng, H. Jiang, J. Yang, J. Zheng, Y. Qin, Z. Li, H. Yu)

**Keywords:** influenza A(H7N9), clinical severity, epidemiology, influenza, viruses, China, respiratory infections

## Abstract

Since March 2013, a novel influenza A(H7N9) virus has caused 3 epidemic waves of human infection in mainland China. We analyzed data from patients with laboratory-confirmed influenza A(H7N9) virus infection to estimate the risks for severe outcomes after hospitalization across the 3 waves. We found that hospitalized patients with confirmed infections in waves 2 and 3 were younger and more likely to be residing in small cities and rural areas than were patients in wave 1; they also had a higher risk for death, after adjustment for age and underlying medical conditions. Risk for death among hospitalized patients during waves 2 and 3 was lower in Jiangxi and Fujian Provinces than in eastern and southern provinces. The variation in risk for death among hospitalized case-patients in different areas across 3 epidemic waves might be associated with differences in case ascertainment, changes in clinical management, or virus genetic diversity.

## Abstract 2

Variation in risk for death might be associated with differences in case ascertainment, changes in clinical management, or virus genetic diversity.

More than 3 years have passed since novel influenza A(H7N9) virus infections were first detected among humans in mainland China (*1*). The first epidemic of human infections occurred in the spring of 2013; 134 cases were laboratory confirmed through September 2013 (control measures in combination with environmental factors led to a lull in incidence in the summer of 2013) (*2*,*3*). However, a second epidemic of infections occurred in the winter of 2013–14 (*4*), and a third epidemic occurred in the winter of 2014–15. A fourth wave is ongoing, and as of March 3, 2016, in mainland China, 730 laboratory-confirmed human cases of influenza A(H7N9) virus infection have been reported, most associated with severe disease; 295 of the infections were fatal. Low pathogenicity of influenza A(H7N9) infections in chickens has been well established (*5*), and most human infections can be attributed to close contact with infected chickens, particularly in live poultry markets (*1*,*6*,*7*).

The objectives of this study were to compare the epidemiology of human cases of influenza A(H7N9) infection across the 3 epidemics and, in particular, to examine whether the severity of disease among hospitalized case-patients has changed over time. To do this, we estimated the risks for death, use of mechanical ventilation, and admission to an intensive care unit (ICU) among hospitalized patients with severe infections caused by influenza A(H7H9) virus.

## Methods

### Source of Data

All laboratory-confirmed cases of avian influenza A(H7N9) virus infection in mainland China are reported to the Chinese Center for Disease Control and Prevention (China CDC) through a national surveillance system. Case definitions, surveillance for identification of cases, and laboratory assays have been described (*8*,*9*). Demographic, epidemiologic, and basic clinical data on each confirmed case-patient were obtained on standardized forms and entered into an integrated database at China CDC. We based our analyses on the version of this database existing on June 15, 2015; we retrieved information about patient age, sex, place of residence, occupation, underlying medical conditions, potential exposure to live poultry, dates of illness onset, hospital admission, ICU admission, mechanical ventilation, death, and recovery or discharge.

### Ethical Approval

The National Health and Family Planning Commission ruled that the collection of data for laboratory-confirmed cases of influenza A(H7N9) virus infection was part of a continuing public health investigation of an emerging outbreak. The study was therefore exempt from institutional review board assessment.

### Statistical Analysis

We analyzed data on the severity of laboratory-confirmed case-patients who were hospitalized for medical reasons (rather than for the sole purpose of isolating them from the community) on the basis of clinical judgment (e.g., those exhibiting complications such as pneumonia). After excluding a small number of case-patients who had mild respiratory symptoms and had been hospitalized only for the purpose of isolation, we estimated the risks of ICU admission, mechanical ventilation, and death after hospitalization (*8*). The number of such cases was small, and inclusion of these cases in analyses did not have any effect on the conclusions. We also excluded case-patients for whom clinical outcomes were not reported from the analysis of severity. We estimated the risk for ICU admission, mechanical ventilation, and death following hospitalization by dividing the number of case-patients who were admitted to ICU, treated with mechanical ventilation, or died by the number of all case-patients with definite clinical outcomes. We derived binomial 95% CIs for each point estimate of severity among hospitalized case-patients.

We examined epidemiologic time-to-event distributions using kernel density methods as described (*9*) and conducted a logistic regression analysis to investigate potential factors affecting the risk for death among hospitalized patients infected with influenza A(H7N9) virus in 3 waves. We estimate adjusted odds ratios and 95% CIs for potential risk factors, including age, sex, place of residence, underlying medical conditions, and time delay from symptom onset to hospital admission. We performed all statistical analyses by using R version 3.0.1 (R Foundation for Statistical Computing, Vienna, Austria).

## Results

**Figure 1:**
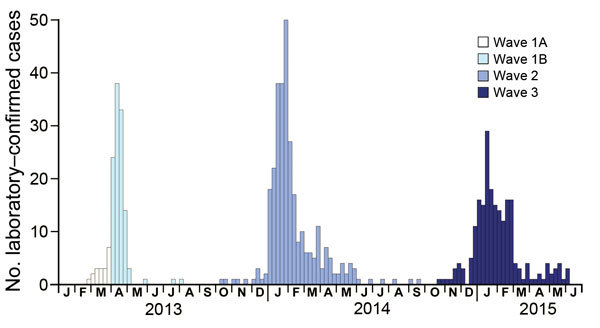
Weekly hospital admissions of human case-patients with laboratory-confirmed influenza A(H7N9) virus infection in 3 epidemic waves, China, 2013–2015.

Three major epidemics of human influenza A(H7N9) virus infections have occurred since the first human case was identified in March 2013: spring 2013, winter–spring of 2013–14, and winter–spring of 2014–15 (Figure 1; Technical Appendix). The median age of confirmed case-patients in each of the 3 epidemics was 61 years, 57 years, and 56 years, respectively. Most patients with laboratory-confirmed cases were men, and a substantial proportion of case-patients had underlying medical conditions (Table 1). Approximately half of the laboratory-confirmed cases in the first wave were detected in municipalities or provincial capital cities such as Shanghai, Hangzhou, and Nanjing; in the second and third waves, most of the case-patients were from smaller cities or rural areas (Table 1; Technical Appendix).

**Table 1:** Characteristics of laboratory-confirmed influenza A(H7N9) cases detected in 3 epidemic waves, China, 2013–2015

Characteristic	No. (%) cases			
	Wave 1A, Jan 2013–Mar 2013, n = 19	Wave 1B, Apr 2013–Sep 2013, n = 115	Wave 2, Oct 2013–Sep 2014, n = 306	Wave 3, Oct 2014–Mar 2015, n = 215
Age group, y				
0–15	1 (5)	6 (5)	19 (6)	17 (8)
16–59	8 (42)	47 (41)	151 (49)	113 (53)
60–74	8 (42)	37 (32)	89 (29)	58 (27)
>75	2 (11)	25 (22)	47 (15)	27 (13)
Median	60	61	57	56
Male sex	13 (68)	81 (70)	212 (69)	154 (72)
Residence*				
Provincial capital or municipality	10 (53)	58 (50)	57 (19)	22 (10)
Other cities	6 (32)	23 (20)	131 (43)	100 (47)
Rural areas	3 (16)	34 (30)	118 (39)	93 (43)
Presence of ≥1 underlying medical condition†	10 (53)	42 (37)	91 (30)	64 (30)
Onset to hospital admission, d				
0–2	2 (11)	12 (10)	41 (14)	32 (18)
3–6	8 (44)	62 (54)	151 (52)	84 (46)
>7	8 (44)	41 (36)	96 (33)	65 (36)
Poultry exposure				
Any exposure to poultry	15 (79)	91 (83)	165 (81)	116 (74)
Occupational exposure to live poultry	1 (5)	6 (5)	21 (7)	22 (10)
Visited live poultry market	12 (63)	62 (54)	132 (61)	105 (67)
Exposure to sick or dead poultry	0 (0)	3 (3)	3 (1)	7 (5)
Exposure to backyard poultry	6 (35)	48 (49)	34 (20)	4 (2)

*For more information, see the online Technical Appendix (http://wwwnc.cdc.gov/EID/article/22/6/15-1752-Techapp1.pdf). †Only underlying medical disorders associated with a high risk for influenza complications were counted here, including chronic respiratory disease, asthma, chronic cardiovascular disease, diabetes, chronic liver disease, chronic kidney disease, immunosuppressed status, and neuromuscular disorders.

**Figure 2:**
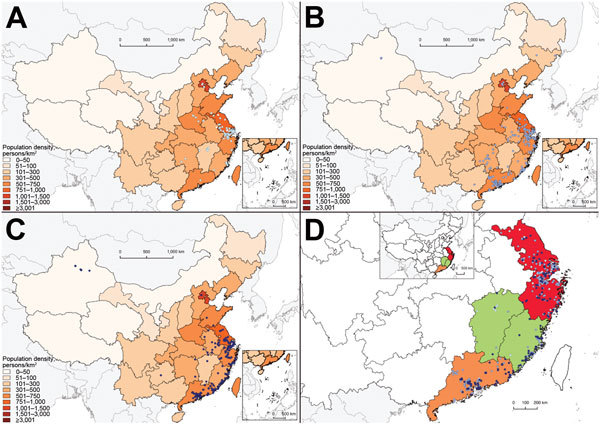
Geographic distribution of human cases of laboratory-confirmed influenza A(H7N9) virus infection, China, 2013–2015. A) Cases detected in wave 1A (white dots) and wave 1B (light blue dots); B) cases detected in wave 2 (medium blue dots); C) cases detected in wave 3 (dark blue dots); D) cases detected in eastern China (red), Jiangxi and Fujian Provinces (green), and Guangdong Province (yellow).

Although human cases in the first epidemic were concentrated in the Yangtze River delta (Figure 2, panel A), human cases in the second and third epidemics occurred across a broader swathe of the country (Figure 2, panels B and C). A separate study that used virus sequence data showed that the viruses in the different parts of China had diverged by the start of the second epidemic, forming 3 separate clades (Figure 2, panel D) (*10*).

**Figure 3:**
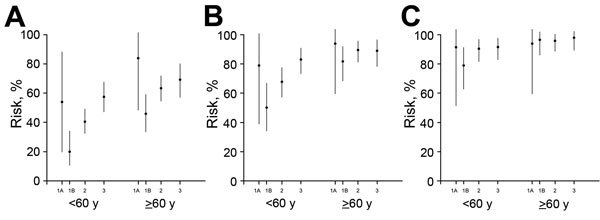
Estimated risk for serious outcomes among patients with confirmed cases of influenza A(H7N9) virus infection hospitalized for medical reasons and 95% CIs, by age and epidemic wave, China, 2013–2015. A) Risk for death; B) risk for death or mechanical ventilation; C) risk for death or mechanical ventilation or intensive care unit admission. The periods covered by waves 1A, 1B, 2, and 3 are shown in Figure 1.

We previously divided the first epidemic into 2 parts—wave 1A for case-patients hospitalized before April 1, 2013, and wave 1B for case-patients hospitalized from April 1 to September 30, 2013—because of higher risks for among case-patients hospitalized before March 31, 2013, the date when the first confirmed human cases of influenza A(H7N9) virus infection were officially announced in China (*11*). We then estimated clinical severity among hospitalized case-patients as measured by hospitalization fatality risk, mechanical ventilation fatality risk, and ICU fatality risk over 3 waves (with wave 1 divided into 2 parts) (Figure 3). Apart from wave 1A, which included mainly retrospective detection of severe cases, some evidence indicated that the severity of hospitalized case-patients increased over time, with statistically significantly higher risk for death among hospitalized patients in wave 3 than for case-patients in wave 1B among those <60 years of age and >60 years of age (Figure 3).

We then examined whether these differences in risk for death could be explained by changes in the characteristics of patients across the 3 waves. In a regression analysis, we found that hospitalized case-patients in wave 2 or 3 had a higher risk for death than those in wave 1B after adjusting for patients’ demographic characteristics and underlying medical conditions (Table 2). The higher risk for death observed in waves 2 and 3 remained significant after further adjustment for patients’ residence and delay from symptom onset to hospital admission (Table 2). Patients >60 years of age had a higher risk for death, and rural patients were less likely to die than urban patients.

**Table 2:** Comparison of risk of death among patients with laboratory-confirmed influenza A(H7N9) virus infection detected in 3 epidemic waves, China, 2013–2015

Characteristic	Laboratory-confirmed H7N9 deaths, adjusted odds ratio (95% CI)	
	Model 1	Model 2
Wave		
1A	4.88 (1.64–14.53)	5.07 (1.67–15.46)
1B	1.00	1.00
2	2.39 (1.46–3.91)	3.48 (2.00–6.06)
3	3.93 (2.30–6.72)	4.84 (2.66–8.80)
Age group, y		
0–15	0.56 (0.05–5.78)	0.50 (0.05–5.43)
16–59	1.00	1.00
60–74	2.05 (1.38–3.04)	2.09 (1.34–3.26)
>75	2.88 (1.75–4.74)	2.49 (1.44–4.30)
Sex		
F	1.00	1.00
M	1.00 (0.68–1.46)	0.94 (0.61–1.45)
Underlying medical conditions		
No underlying medical disorder	1.00	1.00
>1 underlying medical condition*	1.28 (0.88–1.84)	1.22 (0.82–1.81)
Residence		
Residence in a provincial capital or municipality	–	1.00
Residence in other cities§	–	0.52 (0.30–0.89)
Rural residence	–	0.53 (0.31–0.91)
Onset to final hospital admission, d		
0–4	–	1.00
5–7	–	1.21 (0.77–1.90)
>7	–	1.19 (0.72–1.95)

*Only underlying medical disorders associated with a high risk for influenza complications were counted here, including chronic respiratory disease, asthma, chronic cardiovascular disease, diabetes, chronic liver disease, chronic kidney disease, immunosuppressed status, and neuromuscular disorders.

**Figure 4:**
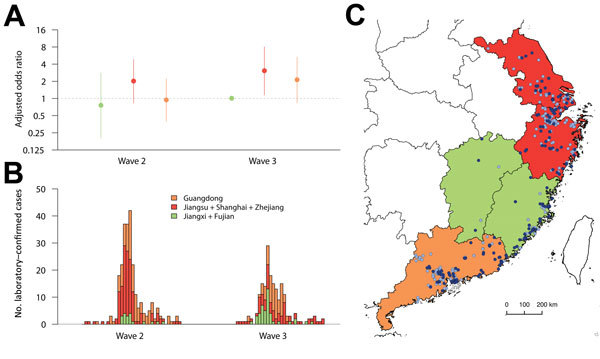
Comparison of risk for death among hospitalized patients with laboratory-confirmed influenza A(H7N9) virus infection detected in 3 areas of China where circulating influenza A(H7N9) viruses might belong to distinct genetic clades, 2013–2015. A) Odds ratios for death, adjusted for age, sex, patient’s residence, underlying medical conditions, and delay from onset to hospital admission; B) symptom onsets of case-patients detected in 3 areas; C) geographic distribution of cases detected in 3 areas. The periods covered by waves 2 and 3 are shown in Figure 1.

We conducted a further analysis to investigate the risk for death among patients in different geographic locations where research suggested that circulating influenza A(H7N9) viruses belonged to different genetic clades (Figure 4) (*10*). We found that hospitalized case-patients detected in Jiangxi and Fujian in wave 3 had a lower risk for death than case-patients reported in eastern China, including Shanghai, Zhejiang Province, and Jiangsu Province, particularly in the third wave, as well as in Guangdong Province in southern China. However, the severity of infection in hospitalized case-patients was similar in patients detected in waves 2 and 3 in Jiangxi and Fujian Provinces (Figure 4).

**Figure 5:**
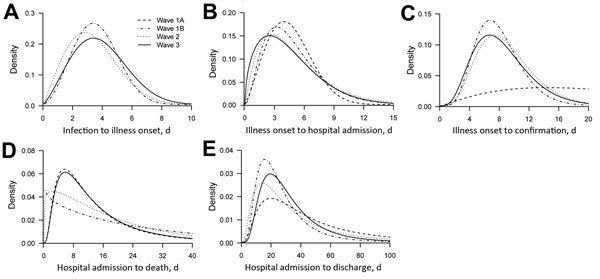
Time to event distributions of influenza A(H7N9) virus infections across different epidemic waves. A) Time from potential exposure to illness onset; B) time from illness onset to hospital admission; C) time from illness onset to laboratory confirmation; D) time from hospital admission to death; E) time from hospital admission to discharge. The periods covered by waves 1A, 1B, 2, and 3 are shown in Figure 1.

We found estimates of the incubation period (Figure 5, panel A) and the time from illness onset to laboratory confirmation (Figure 5, panel C) consistent across the 3 waves. The time from illness onset to hospital admission was relatively shorter in more recent waves than in previous waves (Figure 5, panel B). The mean time from illness onset to laboratory confirmation was 8.0 days in wave 1B, 9.0 days in wave 2, and 8.4 days in wave 3 (analysis of variance, p = 0.44). The time from final hospital admission to death was longer for patients detected in the third wave than for patients from wave 1B and wave 2, whereas the distribution of the interval was similar for wave 3 and 1A (Figure 5, panel D). The time from hospital admission to discharge was generally similar across different epidemic waves (Figure 5, panel E).

## Discussion

In this study, we found some evidence that the estimated risk for severe outcomes in hospitalized patients with influenza A(H7N9) virus infection may have increased in some areas across the 3 epidemic waves over time. Although hospitalized patients in the first part of wave 1 (wave 1A) had more severe cases (perhaps because of ascertainment biases) (*4*), hospitalized patients in the main part of the first wave (wave 1B) generally had less severe cases (Figure 3). The risk for death among hospitalized patients in the second and third waves was higher than the risk for younger and older persons in wave 1B (Figure 3), and this finding could not be fully explained by differences in age, prevalence of underlying medical conditions, or urban/rural residence (Table 2). This difference occurred despite a faster time to admission and similar time to laboratory confirmation of cases (Figure 5).

This observed increase in estimates of severity of cases among hospitalized patients could be real and indicative of an increase in pathogenicity of the virus in humans, or an artifact of case ascertainment biases. In the first hypothesis, apart from potential changes in the virus, an increase in pathogenicity in hospitalized case-patients would also arise if management and treatment of patients differed between the waves (*4*). Infections in the winter in waves 2 and 3 rather than the spring in wave 1B (Figure 1) might be more severe if other pathogens that could cause secondary or co-infections among infected patients were more prevalent. On the other hand, it is possible that prioritized repeating laboratory testing in the early wave of influenza A(H7N9) virus infections and increased laboratory capacity in testing for the virus, particularly among patients with more severe cases over the past 2 years might have led to an artifactual increase in severity of cases among hospitalized patients (*4*). We also observed that a relatively lower proportion of hospitalized patients in the second and third waves were transferred to major regional referral hospitals than in the first wave, which might contribute to a potential increase of clinical severity among hospitalized case-patients if different hospitals were assumed to vary in their capacity for managing these patients.

The relatively lower severity of cases among hospitalized patients estimated in Jiangxi and Fujian Provinces in contrast to the higher severity of hospitalized cases in eastern China and Guangdong Province might be driven by different factors, although the geographic distribution in severity of cases among hospitalized patients was largely consistent with the 3 genetic clades detected in similar areas (*10*). Infections in Jiangxi and Fujian Provinces might be associated with 1 of the 3 clades identified in wave 2 (clade W2-C), whereas the other 2 virus clades originated from provinces in eastern China (clade W2-A) and Guangdong Province (clade W2-B) (*10*), possibly implying a change in virus pathogenesis. Another explanation for the potential increased severity cases among hospitalized patients in these provinces, other than potential viral changes, is that clinical management may have improved in other provinces that acquired more experience in treating these infections. However, this difference may also be an artifact of differential case ascertainment rather than real differences in severity of cases in hospitalized patients, and we did not have access to individual virus sequence data to confirm that each of the cases in Jiangxi and Fujian Provinces was associated with clade W2-C viruses.

Across the 3 epidemics, the declining median age of case-patients might result from population-level behavioral changes in exposure to live poultry, particularly in potential high-risk groups such as the elderly, as indicated in previous studies (*9*,*12*). Population contact with live poultry decreased in influenza A(H7N9) virus–affected and –nonaffected areas after cases were detected in China, although exposure to live poultry in urban and rural areas remained high in the country (*12*). Live poultry markets in China, particularly in cities, have been closing either temporarily or permanently since the first wave in 2013 (*2*,*13*). Some cities severely affected by influenza A(H7N9) virus in the Yangtze River Delta permanently closed all live poultry markets in 2014, which led to a substantial decline in population exposure to the virus and the risk for infection. A relatively higher proportion of rural cases were anticipated in later waves than in earlier waves because contact with backyard poultry instead of commercial live poultry in markets accounted for most poultry exposure for residents of rural and semiurban areas (*14*). The similar geographic dispersion of case-patients in wave 2 and 3 matches the areas with the highest poultry density in eastern and southern China (*15*) and is also consistent with virus genetic findings that new virus clades originating from eastern China in wave 2 might have been well established and become endemic locally in southern China or other areas (*10*). 

Although we have focused on the severity of cases among hospitalized patients, it is also possible to characterize severity in other ways, such as the risk for severe disease among persons with symptomatic influenza A(H7N9) virus infections (*4*,*8*) or the risk for mild and severe disease among all infections. The latter could be estimated if serologic data were available, but few population-based serologic studies of influenza A(H7N9) virus infections have been published (*16*,*17*).

Our study is limited by potential underascertainment of hospitalized case-patients, particularly because of the insufficient capacity of health care facilities to deal with a sudden increased need for diagnosis and treatment of patients with severe cases or a decreasing vigilance on influenza A(H7N9) virus infection in population or health care settings. Ascertainment of hospitalized case-patients could also be potentially affected by changes in clinical management or disease surveillance, particularly of severe acute respiratory infections or unexplained pneumonia through which many cases of influenza A(H7N9) virus infection were detected. However, no major changes were identified in clinical and surveillance practice during the study period. The estimates of the risks of serious outcomes among hospitalized patients could be affected by case ascertainment; limited access to laboratory testing, especially in rural areas; and self-reported exposure data by patients that could be subject to reporting and recall bias. 

We did not have detailed information on clinical management such as oseltamivir use, and therefore we could not explore whether differences in treatment were associated with differences in risk for death. Using a composite endpoint to measure severity of cases in hospitalized patients might provide more information on severe outcomes related to this infection than death only, although use of ventilation and admission to ICUs can be limited by hospital capacity and availability of resources. Inaccuracies in the exact dates of hospitalization and uncertainty about a small proportion (5%) of final outcomes might lead to small biases in our estimates but should not change the overall conclusions of this study.

In conclusion, our study explored the epidemiology of human infections with H7N9 virus in mainland China across 3 epidemic waves in 2013–2015. Laboratory-confirmed H7N9 case-patients were younger and more likely to be from small cities and rural areas in wave 2 and wave 3 than in wave 1. Hospitalized H7N9 patients had an increasing risk for death across 3 waves. The increased risk in waves 2 and 3 might imply a changing pathogenesis associated with genetic clades of H7N9 virus that appeared in later epidemic waves or differences in clinical management in different provinces, although case ascertainment bias could not be ruled out.

Technical Appendix Table shows the number of influenza A(H7N9) cases, deaths, intensive-care-unit admissions, and those on mechanical ventilation reported during epidemic waves in mainland China, 2013–2015, and also a definition of the term residence as used in the study.
