# Supplementary material for: Human Infection with Influenza A(H7N9) Virus during 3 Major Epidemic Waves, China, 2013–2015
Source: Emerg Infect Dis. 2016 Jun;22(6):964–72. doi: 10.3201/eid2206.151752 (PMC4880089; doi:10.3201/eid2206.151752)
Supplement: Technical Appendix — Table shows the number of influenza A(H7N9) cases, deaths, intensive-care-unit admissions, and those on mechanical ventilation reported during epidemic waves in mainland China, 2013–2015, and also a definition of the term residence as used in the study. [file 15-1752-Techapp-s1.pdf]

Article DOI: http://dx.doi.org/10.3201/eid2206.151752

Human Infection with Influenza A(H7N9) Virus during 3 Major Epidemic Waves, China, 2013–2015

Technical Appendix

Technical Appendix Table. Influenza A(H7N9) cases, deaths, intensive-care-unit admissions, and those on mechanical ventilation reported during epidemic waves in mainland China, 2013-2015

	No. (%) by epidemic wave			
Characteristic	1A, N = 19	1B, N = 115	2, N = 306	3, N = 215
Deaths	12 (63)	33 (29)	129 (42)	100 (47)
Intensive-care-unit admission	15 (79)	71 (62)	106 (35)	104 (48)
Mechanical ventilation	14 (74)	57 (50)	92 (30)	90 (42)
Mild case*	1 (5)	9 (8)	33 (11)	27 (13)

*Patients were admitted to hospitals not due to medical reasons.

Definition of Residence Used in the Study

China has a hierarchical administrative system from the top of central government down to provincial, city, county, and town/township governments. Provincial capital cities and municipalities were generally the largest urban areas in the country because of the large population size and stronger economy competency. Other cities in a province are relatively smaller urban areas in terms of population and economy compared with the provincial capital, whereas counties and towns/townships largely consist of rural areas, except for a small urban area where the local government usually is located in each county/town/township. Given the potential difference in exposure to live poultry in “bigger” (provincial capital cities or municipalities) and “smaller” (other medium- or small-size cities) cities, and rural areas (counties/towns/townships excluding urban areas), we therefore divided all places where each patient reported living into 3 categories: 1) provincial capital cities or municipalities, 2). other cities, 3) rural areas, as shown in Tables 1 and 2.
